# Charting the Path Forward for HIV Immune-Based Prevention: Contributions of the Division of AIDS at NIAID

**DOI:** 10.3390/vaccines14060480

**Published:** 2026-05-28

**Authors:** Julia Hutter, M. Patricia D’Souza, Janet M. McNicholl, James R. Lane, Robert W. Eisinger, Cesar Boggiano

**Affiliations:** Division of AIDS, National Institute of Allergy and Infectious Diseases, National Institutes of Health, Bethesda, MD 20892, USA; julia.hutter@nih.gov (J.H.); pdsouza@niaid.nih.gov (M.P.D.); janet.mcnicholl@nih.gov (J.M.M.); laneji@mail.nih.gov (J.R.L.); robert.eisinger@nih.gov (R.W.E.)

**Keywords:** HIV vaccine, immune-based prevention, broadly neutralizing antibodies (bNAbs), germline-targeting immunogens, HIV prevention research, vaccine clinical trials, sequential immunization, HIV immunogen design, pre-exposure prophylaxis (PrEP)

## Abstract

This perspective outlines the ongoing necessity for an HIV vaccine and immune-based prevention strategies in an era of availability of multiple behavioral and pharmacological HIV prevention interventions, including safe and highly effective pre-exposure prophylaxis (PrEP). We describe the approach of the National Institute of Allergy and Infectious Diseases (NIAID), Division of AIDS (DAIDS), based on key scientific progress, critical steps, and persistent challenges in achieving broad and durable immune protection against HIV. We highlight DAIDS coordinated infrastructure, clinical trial networks, and partnerships that enable iterative development and de-risk innovation for these interventions. Finally, we consider implications for trial design and priorities for advancing scalable HIV immune-based prevention.

## 1. Introduction: Closing the HIV Prevention Gap

In the United States, approximately 1.3 million people are living with HIV, with an estimated 38,000 new infections in 2024 and an incidence rate of 13.7 per 100,000 individuals aged 13 years or older, according to the Centers for Disease Control and Prevention. Adults aged 25–44 years accounted for ~65% of diagnoses, with Black and Latino communities, and people living in the Southern U.S., disproportionally affected, and male-to-male sexual contact remaining as the primary transmission route [1].

Although FDA-approved antiretroviral therapy is safe and highly effective, HIV remains a chronic condition without a cure, requiring lifelong treatment to prevent disease progression and achieve viral suppression that eliminates transmission (Treatment as Prevention [TasP], Undetectable=Untransmittable [U=U]). As the acquisition of most HIV infections often occurs in early adulthood, affected individuals may require decades of care, impacting life paths, personal relationships, and overall well-being. Lifetime treatment costs in the United States are estimated to range from $400,000 to $1 million, comparable to some other chronic diseases, such as type 1 diabetes, with a lifetime cost around $500,000 [2,3], underscoring the long-term medical and economic burden. These combined human and economic considerations reinforce and underscore the crucial need for a safe, effective, and accessible HIV vaccine and other immune-based prevention interventions to complement antiretroviral-based prevention and achieve sustained population-level impact.

PrEP has been a central strategy in the HIV prevention toolbox since 2014 [4], but uptake remains suboptimal despite advances in oral, event-driven, and long-acting options, including cabotegravir (CAB-LA) and lenacapavir (LEN). While an estimated 2.2 million individuals in the U.S. could benefit from PrEP in 2025 [5], uptake has increased by only 17% in the U.S. from 2023 to 2024, underscoring that PrEP coverage remains insufficient [6]. Globally, the prevention gap has widened, with new HIV infections now exceeding PrEP initiations by more than twofold [7].

Twice-yearly injectable LEN has demonstrated near-complete efficacy [8,9], with early rollout progressing rapidly relative to prior PrEP modalities [10]. However, current experience with long-acting injectable prevention highlights persistent implementation challenges, including missed or delayed injections due to clinic access, scheduling, and patient-level barriers, which can compromise effectiveness outside of controlled clinical trial settings [11]. These challenges are compounded by structural and economic constraints, including high cost (LEN > $28,000 in the U.S.) [12], limited insurance coverage, regulatory delays, and the need for clinical infrastructure to support parenteral delivery, as well as injection-site reactions and nodules [13]. As a result, uptake remains limited worldwide, with fewer than 3% of eligible individuals currently using CAB-LA, and fewer than 1% using LEN for HIV prevention [14]. These challenges underscore a central advantage of vaccinations and other immune-based interventions; specifically, durable protection that can be delivered at scale, with less dependence on ongoing individual engagement and repeated access to health care facilities.

Current HIV prevention strategies remain heavily dependent on risk recognition and sustained care engagement. Many individuals do not identify themselves as at risk, and stigma and structural barriers have resulted in limited access to prevention services [15,16]. As a substantial proportion of new HIV infections occur outside narrowly defined high-risk groups, achieving population-level impact that is broad, durable, and simple to deliver is essential [7]. The experience of the Contraceptive CHOICE Project offers a useful parallel: when cost, access, and knowledge barriers are removed, people often choose the method that works best for them, with a preference for the safest, most effective, and least user-dependent methods [17].

A safe and effective HIV vaccine and other immune-based prevention strategies would provide scalable, durable, population-level prevention and remain essential to closing the prevention gap. This perspective outlines the scientific strategy for HIV immune-based prevention, the role of DAIDS in advancing it, and key steps that can guide the field forward.

## 2. DAIDS HIV Immune-Based Prevention Path

All HIV preventive strategies share the same goal: to block initial cellular infection and prevent establishment of a lifelong viral reservoir. A safe and effective HIV vaccine and other immune-based prevention approaches must either prevent infection at the portal of entry or rapidly eliminate infected cells before dissemination [18]. The DAIDS approach centers on three linked objectives: induce or deliver broadly neutralizing antibodies (bNAbs); elicit complementary immune responses that can eliminate infected cells; and use iterative clinical testing to refine candidates and down select the most promising approaches.

The last decade has seen rapid advances in technologies and platforms catalyzed by funding from governments, philanthropic organizations, and private investors [19,20], resulting in accelerated development of vaccines, adjuvants, monoclonal antibodies, therapeutics, and diagnostics. Emerging tools, including artificial intelligence (AI), machine learning [21], and synthetic biology [22], are increasingly applied to HIV immunogen design, enabling prediction of epitope structure, antibody–antigen interactions, and iterative optimization [23].

Multiple HIV vaccine strategies, from protein subunits and viral vectors to prime-boost and mosaic approaches, have been evaluated in Phase 2/3 efficacy trials, collectively advancing the understanding of platform design, immunogen selection, and adjuvant choices and, most importantly, how to elicit and assess protective immune responses in humans [24,25]. A key milestone was the RV144 trial, which demonstrated modest, short-lived protection and enabled detailed correlates analyses showing that higher IgG responses to the V1/V2 region of Env (gp120) were inversely associated with HIV-1 acquisition [26]. Subsequent studies (Uhambo, Imbokodo, Mosaico) testing related concepts in different epidemiologic settings and with updated inserts, adjuvants, and platforms showed vaccine-induced binding antibodies were insufficient to confer broad, durable protection in these trials [27,28,29]. Additional evidence suggesting that regional viral characteristics, such as subtype AE in Thailand, may have been a key driver of the modest efficacy observed in RV144 [30]. These observations, together with the lack of protective efficacy of T-cell approaches in humans [31], propelled the field away from binding antibody-based strategies and toward evaluating broadly neutralizing antibodies (bNAbs), both as passively delivered agents and as targets for next-generation immunogen design. Given the diversity of HIV globally and in the U.S. [32], neutralizing antibodies with breadth to cover multiple clades of HIV will be needed.

The DAIDS-sponsored Antibody Mediated Prevention (AMP) efficacy trials showed that a single broadly neutralizing antibody (bNAb), VRC01, prevented infection from susceptible HIV strains across geographical regions and modes of HIV transmission [33], establishing proof of principle for antibody-mediated prevention. These findings provide a strong rationale supporting the concept that sufficiently high neutralizing antibody titers can protect against HIV if they can be induced or delivered in a durable way and with sufficient breadth to cover circulating virus strains [34]. Several DAIDS-funded clinical trials of passively administered bNAbs are ongoing, focusing on bNAbs with longer half-lives, greater breadth, and potency, in order to define the optimal combination for future efficacy trials. If efficacious, this approach could provide individuals with an additional choice between bNAb-based and antiretroviral (ARV)-based PrEP, both delivered as twice-yearly injections, with differing side-effect and resistance profiles. Moreover, these trials inform the HIV vaccine and immune-based prevention field by identifying an ideal combination of bNAbs and targets for immunogen design.

**Figure 1:**
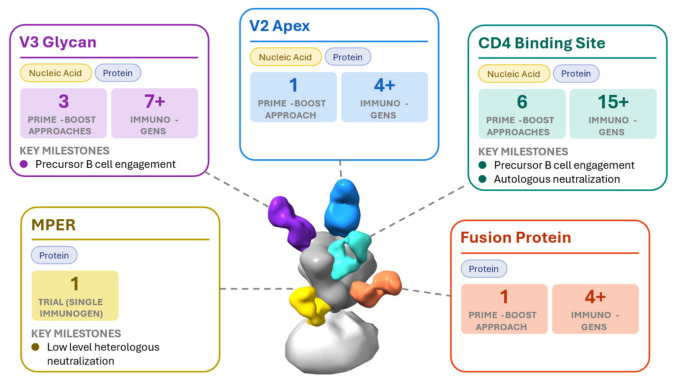
DAIDS Supported Immunogen Approaches to Elicit bNAbs to Key Env Epitopes. The center depicts HIV-1 Env (dark gray) with colored protrusions representing bNAbs bound to their respective targets: the CD4 binding site, V2, V3 glycan, MPER, and fusion peptide. Light gray structure represents the viral lipid membrane. Nucleic acid-based vaccines include DNA and mRNA. Protein-based include adjuvanted Env, Env subunits, and peptides. For each epitope, the left box represents the number of strategies to induce bNAbs and the right box the number of immunogens under evaluation.

Inducing bNAbs against HIV-1 presents enormous challenges due to the extensive genetic variability of HIV, its glycan shielding, conformational masking of epitopes on Env, and ultimately, the need for durable high antibody levels to achieve protection against HIV acquisition [35]. Researchers have identified at least five major conserved Env sites that are targeted by bNAbs: the CD4 binding site (CD4bs); V3 glycan; V2 apex; membrane proximal external region (MPER); and fusion peptide [35] [Figure 1]. Since HIV mutates rapidly, a successful vaccine or other immune-based prevention approach will likely need to target multiple bNAb specificities to achieve breadth across global variants [36].

Germline-targeting (GT) offers a promising strategy to induce bNAbs by activating rare B cell precursors and guiding their maturation toward bNAb production [35,37]. The GT approach is based on the rational design of priming immunogens engineered to bind and specifically recruit rare precursor bNAb B cells. The priming phase activates these precursor cells, selects for bNAb-like features, and generates immunological memory. This initial response is subsequently driven toward maturation through sequential heterologous boosters that progressively resemble native HIV envelope structures, a process described as “shepherding” [35]. Each successive booster, including shaping and polishing immunogens, guides B-cell affinity maturation and expands the breadth, durability, and titer of the antibody response. As multiple immunizations will be required to shepherd bNAb development [38], strategies to reduce the number of injections and clinic visits, such as co-delivery of immunogen cocktails and development of pulsatile-release technologies, will be important for feasibility and uptake.

Significant progress in this field has been demonstrated through a series of DAIDS-sponsored preclinical and clinical studies. For example, immunogens designed to target the CD4 binding sites (CD4bs) have successfully activated and expanded bNAb precursors in both animal models and humans [35]. Studies using adjuvanted protein and synthetic nucleic acid-based platforms have shown that germline-targeting immunogens, such as eOD-GT8, whose antigen design and development were co-funded by NIAID, can engage and expand VRC01-class B cells in a high proportion of vaccinated individuals across diverse geographic settings [38,39,40]. Similar approaches have been evaluated using sequential boosting immunogens designed to further refine and mature these responses toward increased breadth. Also, membrane-bound Env trimer immunogens delivered via mRNA have been shown to induce more potent neutralizing antibody responses than soluble trimers [41]. Studies of stabilized CH505-derived Env trimer immunogens, formulated with adjuvants, have demonstrated safety and the ability to elicit antigen-specific antibody responses, including early CD4bs–directed responses consistent with activation of bNAb precursor lineages [42]. Observations of adverse events, such as transient hypersensitivity reactions in some recipients of mRNA-based products, have prompted reformulation efforts [43]. These and other mRNA, DNA, and protein-based vaccines and immune-based approaches continue to be actively evaluated in ongoing clinical studies. Beyond immunogen design alone, the magnitude and durability of HIV vaccine-induced antibody responses may also depend on how the antigen is delivered and how innate and germinal center pathways are engaged. Strategies such as escalating-dose regimens, targeted TLR-based adjuvant stimulation, and prolonged antigen persistence may therefore help improve bNAb induction and maturation and support iterative evaluation of immunogens, adjuvants, and regimen strategies within the DAIDS Discovery Medicine framework.

Additional bNAb targets include the V3 glycan and V2 apex, which require long CDRH3 regions and are rare in the naïve B cell repertoire [35]. These precursors have been induced in animal models [23] and are now being tested in clinical trials [44]. MPER-directed immunogens using peptide-liposome priming were the first to show that potent bNAbs can be induced in humans on a timescale much shorter than natural infection, albeit at low levels [43,45,46]. Efforts are ongoing to broaden and strengthen these immune responses with new boosting immunogens and formulations to improve safety and tolerability [46]. Vaccine candidates targeting the fusion peptide have shown protective efficacy in animal models [47,48] and are under clinical evaluation with next-generation immunogens under development.

Progress has been made in designing priming immunogens that activate rare B-cell precursors and developing boost immunogens that drive the maturation of bNAbs [35]; however, no immunization regimen has yet achieved the breadth and potency needed for consistent protection against HIV acquisition. High titers of bNAbs remain a prerequisite, and the AMP trials and nonhuman primate studies underscore the need for complementary vaccine and immune-based strategies, including adjuvants, to engage additional arms of the immune system that may reduce the threshold of bNAbs needed for protection. In addition to neutralization, Fc-mediated antibody effector functions, including ADCC and other responses that engage innate effector cells such as NK cells, may contribute to antiviral activity, while cellular immune responses may help eliminate infected cells before reservoir establishment. Consistent with this broader view, DAIDS supports parallel approaches that include adjuvant and regimen strategies to shape antibody quality and durability, as well as vaccine candidates designed to elicit cellular immunity, including CMV-vectored vaccine candidates [49] and approaches targeting conserved regions of the HIV proteome [50,51,52].

An important related question is which immunologic endpoints should guide advancement of HIV vaccine candidates. Although no validated immune surrogate of protection yet exists for active HIV vaccination, a practical framework includes engagement of the intended naïve B-cell precursors, progression of those lineages through sequential boosting, and induction of serum neutralizing activity of sufficient magnitude, breadth, and durability against diverse tier 2 viruses [53], benchmarked where possible to protective titers inferred from passive antibody studies. Additional desirable endpoints include durable memory B-cell responses, sustained germinal center activity, mucosal antibody, Fc-mediated effector functions, and cellular immune responses capable of recognizing and eliminating infected cells.

Host factors also shape immune-based prevention strategies. Systems analysis of HIV vaccine candidate trials shows that early innate responses influence downstream immunity [54]. Particularly in the RV144 trial, type I interferon signaling was activated within 1 day of vaccination, directly shaping subsequent humoral responses measured 6.5 months later, demonstrating that peak transcriptional responses in interferon pathways were associated with favorable antibody functions [54]. Preclinical evaluation of a CMV-vectored HIV vaccine candidate protecting ~55% of vaccinated macaques identified an innate immune signature featuring IL-15 pathway activation as a key correlate of vaccine efficacy [55]. Broader systems vaccinology studies have identified shared transcriptional signatures that predict antibody magnitude and durability [56,57]. Additionally, reactogenicity and safety vary with demographic and metabolic factors, including sex, age, and body mass index [58]. Modifiable external factors—including environmental exposures, nutrition, and the microbiome—may further influence immune responses and tolerability; improved understanding of these factors, including through computational immunology and integration into clinical trials, may help optimize HIV prevention strategies.

The landscape of HIV prevention is changing due to the success of antiretroviral-based prevention, which impacts the design and conduct of future vaccine and immune-based efficacy trials. These studies may require substantially larger sample sizes and the incorporation of innovative methodologies, such as recruiting study participants who are unable or unwilling to use PrEP. Ethical considerations regarding the use of control groups remain important and may also be addressed through approaches such as counterfactual trial designs [59].

Another strategy to advance our understanding of bNAb induction is to evaluate vaccine candidates in people living with HIV. These studies allow direct measurement of bNAb lineage evolution, and effects on viral load and the reservoir can be directly assessed, particularly during analytical treatment interruptions (ATI) [60]. In the context of bNAb-mediated ATI, recent studies have shown that pre-existing stem-like CD8^+^ T cells (e.g., TCF-1^+^ progenitor populations) are associated with improved viral control and delayed rebound and can undergo robust expansion and differentiation into effector cells at the time of viral rebound. This highlights both their baseline functional capacity and their dynamic recall response as key determinants of post-treatment control [61,62]. Importantly, the data generated through these ATI studies may also meaningfully advance HIV therapy and cure research.

In summary, advances in immunology, structural biology, vaccine platforms, and computational design have accelerated rational HIV vaccine development. Proof of principle for antibody-mediated prevention has been established, but consistent protection will likely require multiple epitopes (2–3), complementary immune responses, and feasible trial designs in the era of effective PrEP. Germline-targeting, lineage-based sequential immunization, and structure-guided immunogen design together define complementary strategies that show promise for multiple conserved Env regions. Key milestones must be achieved to go from engagement of B-cell precursor to successful affinity maturation toward potent and durable neutralization [35]. Addressing these challenges will require coordinated leadership and strong global partnerships.

## 3. The Role of DAIDS in the Development of HIV Immune-Based Prevention Approaches

The National Institutes of Health (NIH) provides the majority of global funding for HIV prevention research and development (R&D), contributing approximately 64% of total investment worldwide [63]. In 2024, NIH allocated approximately $591 million to support HIV immune-based prevention [64]. These sustained investments underscore NIH’s central role in advancing HIV prevention, with NIAID leading efforts from basic discovery through clinical evaluation and implementation research. NIAID’s new vision for HIV control includes maintaining a strong R&D pipeline in the areas of therapeutic and preventive strategies [65]. NIAID will concurrently expand research on how to implement tools to control HIV that can be applied to real-world settings and achieve progress in ending the HIV epidemic.

Within NIAID, DAIDS supports a comprehensive global research portfolio spanning HIV pathogenesis, HIV coinfections and comorbidities [66] and the development of safe and effective treatment and prevention strategies. For immune-based prevention, DAIDS provides a coordinated structure that links discovery science, translational development, manufacturing, clinical testing, and data analysis. This structure is especially important for early-stage vaccine development, where scientific uncertainty and financial risk are high, and industry participation is limited.

DAIDS-supported networks have generated pivotal findings from over 200 studies of vaccines and monoclonal antibodies, including six efficacy trials conducted at over 70 sites across four continents. Within DAIDS, the Vaccine Research Program (VRP) coordinates the discovery, development, and clinical evaluation of immune-based HIV prevention approaches. Supported through grants, cooperative agreements, and targeted contracts, DAIDS de-risks early-stage interventions by supporting critical proof-of-concept studies and absorbing early financial and operational risk and enabling iterative advancement and further evaluation, often through public–private and academic partnerships. VRP activities span preclinical design, translational development, GMP manufacturing, and clinical testing within an integrated, adaptive pipeline coordinated by the HIV Vaccine Trials Network (HVTN) [67] Leadership and Operations Center, and conducted in collaboration with the Advancing Clinical Therapeutics Globally (ACTG) [68], the International Maternal Pediatric Adolescent AIDS Clinical Trials Network (IMPAACT) [69], and the HIV Prevention Trials Network (HPTN) [70].

The Networks Clinical Trials Units provide core site infrastructure, with DAIDS oversight ensuring regulatory compliance and performance. Site selection is competitive and guided by epidemiology, target populations, and clinical capacity. DAIDS regulatory sponsorship, safety monitoring, and pharmaceutical operations further ensure participant safety, data integrity, and efficient management and distribution of investigational products across trials. Ethical conduct and community engagement are integral, supported by community advisory boards, institutional review boards, and culturally appropriate informed consent processes.

**Figure 2:**
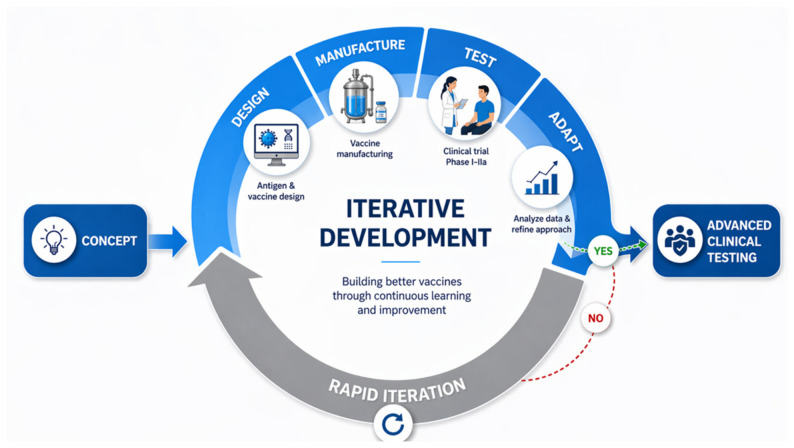
Iterative HIV vaccine development. Stages from concept through design, manufacture, and testing of vaccine candidates are shown. Decisions to change or adapt influence whether to iterate or proceed to advanced testing.

With the shift toward bNAb–inducing immunogens and advances in B-cell biology, immune modulators, and delivery platforms, DAIDS and HVTN established the Discovery Medicine Program [71] [Figure 2]. The program emphasizes rapid, iterative assessment of safety and immunogenicity across immunogens, adjuvants, and delivery platforms. Standardized protocols, central assay plans, and harmonized statistical approaches enable efficient comparison across studies and inform immunogen design, dosing, and regimen strategies. The Discovery Medicine “300 series,” initiated in 2021 [71], has since included more than a dozen studies evaluating multiple immunogen–adjuvant combinations in adults, including people living with HIV, with additional studies in development as data emerges from ongoing trials.

Following discussions with the FDA in 2025, DAIDS and HVTN implemented an umbrella protocol approach in which protocols prospectively define priming immunogens and conditional boosting strategies based on predefined immune criteria, maintaining regulatory oversight while reducing timelines to evaluate sequential immunogen schemas and the number of investigational new drug applications.

The HVTN Laboratory Center provides standardized Good Clinical Laboratory Practice (GCLP)-compliant immunologic and virologic assays, enabling reliable comparisons across studies and platforms. Unique assays also distinguish vaccine-induced seropositivity from HIV infection in trial participants. These standardized approaches inform data-driven decisions on product advancement and often become field-wide standards through the open sharing of reagents and standard operating procedures. In parallel, DAIDS coordinates laboratory oversight across DAIDS vaccine, therapeutics, and prevention trials, harmonizing quality processes and data comparability across clinical trial networks.

The HVTN Statistical and Data Management Center leads trial design, data management, and statistical analysis, ensuring data quality and consistency across studies. DAIDS provides additional oversight and cross-network coordination to support data integrity, reporting, and regulatory compliance.

Collectively, this coordinated ecosystem positions DAIDS to advance HIV immune-based prevention, including vaccines. By linking discovery, translational research, and clinical evaluation with strong partnerships across academia, industry, and communities, DAIDS provides a comprehensive framework advancing promising immune-based candidates toward effective HIV prevention.

## 4. Conclusions: The Path Forward

The HIV prevention landscape is at an inflection point. While highly effective biomedical interventions, including PrEP and TasP, have significantly advanced prevention efforts, their population-level impact remains constrained by access, adherence, cost, and structural barriers. To overcome these limitations, a scalable, population-level immune-based intervention such as a safe, effective, and durable HIV vaccine remains essential to close the HIV prevention gap.

Advancing this goal will require a deliberate, coordinated approach that integrates scientific innovation across both preclinical and clinical domains with implementation foresight:

First, continued prioritization of rational immunogen design, building on advances in germline-targeting and lineage-based approaches directed at conserved Env epitopes, remains essential. Key research milestones include engagement of naïve B-cell precursors and demonstration of affinity maturation along bNAb lineages, with the aim of achieving potent, heterologous neutralization. These efforts should be iteratively tested in robust preclinical models and clinical evaluation frameworks, such as the Discovery Medicine program, to identify the most promising candidates.

Second, advancing an HIV vaccine candidate(s) toward efficacy testing will likely require combination approaches that integrate multiple targets and complementary immune strategies. Protective efficacy will depend on coordinated induction of bNAbs along with cellular immune responses capable of eliminating HIV-infected cells. This will require investment in platforms that enable multi-component regimens, optimized delivery systems, and enhanced durability while minimizing regimen complexity.

Third, advancing the field will require coordinated integration of systems immunology, artificial intelligence, and data science. Leveraging large-scale, cross-trial datasets to identify predictive signatures of immunogenicity, durability, and safety can inform immunogen refinement, optimize trial design, and guide study participant selection while accounting for host factors. Embedding these approaches into clinical development pathways will accelerate and increase the likelihood of success of developing a safe and effective immune-based intervention(s).

Fourth, clinical trial paradigms must continue to evolve in the context of highly effective existing HIV prevention tools. Innovative designs—including counterfactual frameworks, hybrid prevention efficacy studies, and evaluations among people living with HIV—will be essential to generate interpretable efficacy signals while upholding rigorous bioethical standards. At the same time, strengthening clinical trial infrastructure and ensuring meaningful engagement of populations at risk will be critical not only for trial conduct but also for eventual implementation success.

Fifth, sustained progress will depend on robust partnerships and long-term sustained investment. DAIDS is uniquely positioned to support this effort by coordinating across government, academia, industry, and affected communities, while de-risking early-stage development and coordinating scientific alignment across the field. Equally important is maintaining a strong commitment to reaching populations most affected by HIV and ensuring that advances in immune-based prevention are accessible, acceptable, implementable, and scalable.

Importantly, investments in HIV immune-based prevention research extend well beyond HIV/AIDS research. The scientific insights, platforms, and technologies emerging from these efforts are already driving broader advances in immunology and novel biomedical interventions, contributing to broader advances in treatment and prevention of other infectious diseases, cancer, and autoimmune disorders.

In summary, the path forward is not defined by a single breakthrough, but by building on the extensive knowledge foundation in this field and an integrated, coordinated approach that aligns discovery, translational science, clinical evaluation, and implementation science. With sustained commitment, scientific rigor, and broad public–private collaborations, the development of effective HIV immune-based prevention strategies, including a vaccine, remains an achievable and essential goal.

## Data Availability

No primary data were produced for this work.

## Abbreviations

Acronym	Definition
ACTG	Advancing Clinical Therapeutics Globally
AI	Artificial Intelligence
AMP	Antibody Mediated Prevention
ARV	Antiretroviral
ATI	Analytical Treatment Interruption
bNAbs	Broadly Neutralizing Antibodies
BMI	Body Mass Index
CAB-LA	Cabotegravir Long-Acting
CD4bs	CD4 Binding Site
CDC	U.S. Centers for Disease Control and Prevention
CMV	Cytomegalovirus
DAIDS	Division of AIDS (NIAID/NIH)
DNA	Deoxyribonucleic Acid
Env	Envelope (HIV Envelope Glycoprotein)
FDA	U.S. Food and Drug Administration
GCLP	Good Clinical Laboratory Practice
GMP	Good Manufacturing Practice
GT	Germline Targeting
HIV	Human Immunodeficiency Virus
HPTN	HIV Prevention Trials Network
HVTN	HIV Vaccine Trials Network
IgG	Immunoglobulin G
IMPAACT	International Maternal Pediatric Adolescent AIDS Clinical Trials Network
LEN	Lenacapavir
mRNA	Messenger Ribonucleic Acid
MPER	Membrane Proximal External Region
NIAID	National Institute of Allergy and Infectious Diseases
NIH	National Institutes of Health
NK	Natural Killer (cells)
PrEP	Pre-Exposure Prophylaxis
R&D	Research and Development
TasP	Treatment as Prevention
U=U	Undetectable = Untransmittable
V1/V2	Variable Regions 1 and 2 of HIV Envelope
V2 apex	Variable Region 2 Apex
V3 glycan	Variable Region 3 Glycan Site
VRP	Vaccine Research Program (DAIDS/NIAID)
